# Using the COM-B framework to elucidate facilitators and barriers to COVID-19 vaccine uptake in pregnant women: a qualitative study

**DOI:** 10.1186/s12884-023-05958-y

**Published:** 2023-09-06

**Authors:** Lynsey Patterson, Emma Berry, Carole Parsons, Bronagh Clarke, Alison Little, Jillian Beggs, Antony Chuter, Tracy Jackson, Yingfen Hsia, Hannah McGrath, Catherine Millman, Siobhan Murphy, Declan T. Bradley, Sarah Milligan

**Affiliations:** 1 Public Health Agency, Belfast, UK; 2 Centre for Public Health, Queen’s University Belfast, Belfast, UK; 3 School of Psychology, Queen’s University Belfast, Belfast, UK; 4 School of Pharmacy, Queen’s University Belfast, Belfast, UK; 5 PPI, Usher Institute, University of Edinburgh, Edinburgh, UK; 6 Centre for Neonatal and Paediatric Infection, St George’s University of London, London, UK

**Keywords:** COVID-19 vaccination, Pregnancy, Qualitative, Facilitators, Barriers, COM-B

## Abstract

Since April 2021, COVID-19 vaccines have been recommended for pregnant women. Despite this, COVID-19 vaccine uptake in this group is low compared to the non-pregnant population of childbearing age. Our aim was to understand barriers and facilitators to COVID-19 vaccine uptake among pregnant women in Northern Ireland using the COM-B framework, and so to make recommendations for public health interventions. The COM-B proposes that human behaviour is influenced by the extent to which a person has the capability, opportunity, and motivation to enact that behaviour. Understanding the factors underpinning behaviour through this lens helps discern what needs to change to change behaviour, therefore supporting the development of targeted interventions.

This study consisted of eight semi-structured interviews with new/expectant mothers who did not receive a COVID-19 vaccine dose while pregnant since April 2021, and a focus group with five participants who received at least one COVID-19 vaccine dose while pregnant. Interview and focus group data were analysed using semi-deductive reflexive thematic analysis framed by a subtle realist approach. The COM-B was used to categorise codes and subthemes were developed within each COM-B construct.

Within Psychological Capability, subthemes captured the need for consistent and reliable COVID-19 vaccine information and access to balanced and jargon-free, risk–benefit information that is tailored to the pregnant individual. The behaviour/opinions of family, friends, and local healthcare providers had a powerful influence on COVID-19 vaccine decisions (Social Opportunity). Integrating the COVID-19 vaccine as part of routine antenatal pathways was believed to support access and sense of familiarity (Physical Opportunity). Participants valued health autonomy, however experienced internal conflict driven by concerns about long-term side effects for their baby (Reflective Motivation). Feelings of fear, lack of empathy from healthcare providers, and anticipated guilt commonly underpinned indecision as to whether to get the vaccine (Automatic Motivation).

Our study highlighted that the choice to accept a vaccine during pregnancy generates internal conflict and worry. Several participants cited their concern was primarily around the safety for their baby. Healthcare professionals (HCPs) play a significant part when it comes to decision making about COVID-19 vaccines among pregnant women. HCPs and pregnant women should be involved in the development of interventions to improve the delivery and communication of information.

**Supplementary Information:**

The online version contains supplementary material available at 10.1186/s12884-023-05958-y.

## Background

Vaccination has been the cornerstone in the management of several major public health threats throughout history [1]. When the SARS-CoV-2 virus emerged and the COVID-19 pandemic was declared in March 2020, vaccine development and approval was prioritised. In December 2020, roll-out commenced to high-risk population groups, which excluded pregnant women because of a lack of safety data given their exclusion from clinical trials, which is standard practice [2]. However, emerging evidence showed that pregnant women with COVID-19 had a higher risk of severe illness, pre-eclampsia, preterm birth, and stillbirth compared to non-pregnant women [3–6].

On 16^th^ April 2021 the Joint Committee on Vaccination and Immunisation (JCVI) advised that all pregnant women should be offered the COVID-19 vaccine at the same time as their general age cohort. Endorsement of this recommendation by the Royal College of Obstetricians and Gynaecologists (RCOG) and the Royal College of Midwives (RCM) followed on the 22nd of July 2021. On 16 December 2021, based on growing evidence of risks of infection during pregnancy, JCVI strengthened their recommendation to recognise pregnant women as a priority clinical risk group for COVID-19 vaccination [4, 5, 7]. Despite this, vaccine uptake in pregnant women was lower than uptake in the general cohort of women of childbearing age [5, 8–10]. Uptake was also lower among younger pregnant women and those in the most deprived areas [5, 8]. This is despite evidence showing that COVID-19 vaccines were a safe and effective way to reduce the risk of severe disease resulting from infection during pregnancy [5, 11–15].

The term ‘vaccine hesitancy’ has been used to describe a delay in acceptance or refusal of vaccination despite availability of vaccination services [16]. In 2019, it was recognised by the World Health Organization as one of the top 10 threats to global health [17]. Those who are hesitant are typically unsure about getting a vaccine, and form a distinct group to those who strongly object to getting a vaccine, who can be considered vaccine resistant (typically a smaller minority) [18]. Understanding the reasons for vaccine hesitancy among pregnant women can help public health professionals and policy makers adapt their messaging to support women to make informed decisions about vaccines.

The aim of this study was to gain an understanding of the behavioural determinants (barriers and motivators) of COVID-19 vaccination in pregnancy and consider how to support women to make informed decisions about getting a COVID-19 vaccine. This study provides a novel contribution to the existing qualitative evidence in this area by using a theoretical model that is recognised and utilised by Public Health researchers, which enhances the applicability of the findings. To support a structured and theoretically robust investigation of the factors which support or impede vaccine uptake in this population, the Capability, Opportunity, and Motivation model of Behaviour (COM-B) [19] was used. The COM-B model proposes that when a person can perform the behaviour (capability, e.g., knowledge and self-efficacy), when the environment that enables the behaviour is satisfied (opportunity, e.g., time, resource, and social nudges), and when a person feels motivated to engage (motivation, e.g., perceived benefits/value), then behaviour change is more likely [19]. The COM-B model is increasingly used by Public Health professionals and researchers due to its utility in applied health behaviour research. The COM-B model is particularly useful as it conceptually fits within a multi-level model called the Behaviour Change Wheel (BCW) [19], which helps to identify intervention functions that are evidenced to target the capability, opportunity, and motivation factors that underpin the behaviour under study. The BCW also facilitates identification of policy change that may be needed to support the delivery and sustainability of selected interventions. The WHO Tailoring Immunisation Programme (TIP) resource [20] demonstrates the application of the COM-B model and BCW in the context of vaccination behaviour and offers a staged guide as to how interventions can be developed. This research aims to provide findings that can be engaged with in an applied way, with further insights gleaned from the TIP resource [20].

## Methods

### Ethics statement

Ethics approval was obtained from the NHS Health Research Authority, London – Fulham Research Ethics Committee (REC reference: 22/PR/0531). Informed consent was obtained at the recruitment stage and at the start of the interviews or focus group and participants could withdraw at any stage (including after participation).

### Design and setting

The study was conducted in Northern Ireland. The study consisted of eight semi-structured interviews with individuals who did not receive a COVID-19 vaccine dose while pregnant in 2021. The sample was weighted towards those who may consider a COVID-19 vaccine in the future (vaccine hesitant), using a screening questionnaire, with two interviews conducted among those who had no intention of getting vaccinated in the future. The analyst team were blinded to participant intentions. To gather perspectives of participants who received at least one COVID-19 vaccine dose during pregnancy, to elicit additional insights, one focus group with five individuals who had received at least one COVID-19 vaccine dose while pregnant in 2021 was conducted. We used two different approaches according to vaccination status to avoid any unintended consequences of vaccine hesitant women being exposed to strong anti-vaccination sentiment.

A market research company, accredited under the Interviewer Quality Control Scheme (https://iqcs.org) and certified to ISO 20252 and ISO 27001 standards, was commissioned to assist with recruitment, interviews and the transcription process.

### Materials

A screening questionnaire was used to confirm eligibility and consent to participate in the study. Interview and focus group topic guides were adapted from a previous study which had been developed with the involvement of maternity service users [21] [see Additional files 1 and 2]. These were reviewed by the Data and Connectivity: COVID-19 Vaccines Pharmacovigilance (DaCVaP) patient and public involvement and engagement (PPIE) team.

### Participants

Online recruitment, using snowball sampling, was conducted during May and June 2022 by a panel of trained qualitative recruiters who have built up large networks of contacts over time. Online recruitment was chosen as guidelines at the time suggested limiting social contacts for the most vulnerable groups. A financial incentive of £30 (for interview participation) or £35 (for focus group participation) or a “one4all” voucher or charity donation of equivalent value were offered. Eligibility and vaccine behaviour were determined through the screening questionnaire. Inclusion criteria were new/expectant mothers aged 18—45 years who had either: (a) had a pregnancy since April 2021 (aligned to the JCVI recommendation) or (b) were known to be pregnant at the time of recruitment. Recruitment was targeted to ensure representation across broad age groups (18–34 and 35–49 years), health and social care trust area (a geographical area), and socioeconomic status [18] (see Table 1). Health and social care trust area and socioeconomic status were based on the participants area of residence. All participants were of white ethnicity. For three of the participants this was their first pregnancy, and seven of the eight had received a vaccine for influenza or pertussis. The focus group consisted of five participants who had received at least one dose of the COVID-19 vaccine while pregnant (see Table 2). All participants in the focus group had at least one previous pregnancy. Those who had taken part in any market research in the last 12 months or who had family or close friends involved in market research or the vaccine programme were excluded.

**Table 1:** Socio-demographic characteristics of the study population for in-depth interviews

	**Number**	**%**
**Participants (total)**	8	
**Age group**		
18–34	2	25
25–29	2	25
30–34	3	37.5
35–45	1	12.5
**Social grade**		
Higher & intermediate managerial, administrative, professional occupations	1	12.5
Supervisory, clerical & junior managerial, administrative, professional occupations	3	37.5
Skilled manual occupations	2	25
Semi-skilled & unskilled manual occupations, Unemployed and lowest grade occupations	2	25
**Health and Social Care Trust**		
Belfast	2	25
Northern	2	25
Southern	2	25
South-Eastern	0	0
Western	2	25
**First pregnancy**		
Yes	3	37.5
No	5	62.5
**Vaccinated against influenza / pertussis during pregnancy**		
Yes	7	87.5
No	1	12.5

**Table 2:** Socio-demographic characteristics of the study population for the focus group

	**Number**	**%**
**Participants (total)**	5	
**Age group**		
18–34	4	80
35–49	1	20
**Social grade**		
Higher & intermediate managerial, administrative, professional occupations	3	60
Supervisory, clerical & junior managerial, administrative, professional occupations	1	20
Skilled manual occupations	1	20
Semi-skilled & unskilled manual occupations, Unemployed and lowest grade occupations	0	0
**Health and Social Care Trust**		
Belfast	0	0
Northern	0	0
Southern	2	40
South-Eastern	1	20
Western	2	40

### Data collection

A female researcher, trained in qualitative methods, conducted the fieldwork for the interviews and focus group between May and June 2022. Each interview lasted approximately 45 min, and the focus group lasted approximately 90 min. Both the interviews and focus group were conducted online. Interviews and focus group recordings were transcribed verbatim and sent to the project team for independent analysis.

### Analysis

Interview and focus group data were analysed separately by LP (Specialty Registrar and lecturer in Public Health) and EB (Health Psychology Lecturer) using semi-deductive reflexive thematic analysis [22], framed by a subtle realist approach [23]. All parts of the analysis were carried out using Microsoft Excel and Word. In line with Braun and Clarke’s [22] six-step process, lead analysts LP and EB each coded four of the in-depth interviews in MS Word, which was subsequently cross-checked by the other analyst for interrater reliability. LP and EB met weekly during the coding phase to review codes, and agree on the removal, addition, or amalgamation of codes. MS Excel facilitated the further reflective reviewing and organisation of codes. Both LP and EB contributed to the analysis of the focus group transcript.

Initial coding was inductive, and codes were subsequently aligned under the COM-B thematic framework, where they were deemed to conceptually fit [24]. This approach has been successfully applied in previous work exploring the facilitators and barriers to social distancing among young people [24]. Moreover, deductive mapping of codes and subthemes to the COM-B can facilitate the identification of interventions using the Behaviour Change Wheel [19] for public health professionals, which aligned with the aims of this study. ‘Parent’ codes were determined and were used to develop subthemes that remained bound to the source code but provided a higher degree of abstraction. A coding tree was developed to ensure that the subthemes captured the codes and were valid reflections of the data. Thoughts and observations were noted throughout the analysis and perspectives and interpretations were discussed. With the pragmatic constraints on recruitment, we cannot ascertain that data saturation was achieved. However, recurring prominent codes were noted by the point at which the final interviewee transcripts were coded, and any new codes extracted were modest and occurred within individual transcripts. Moreover, data coded for the focus group corroborated the codes and subthemes extracted from the interview data.

Participant demographic/health information was analysed using frequency statistics.

The findings were reported in line with the COnsolidated criteria for REporting Qualitative research (COREQ) [25].

## Results

**Fig. 1:**
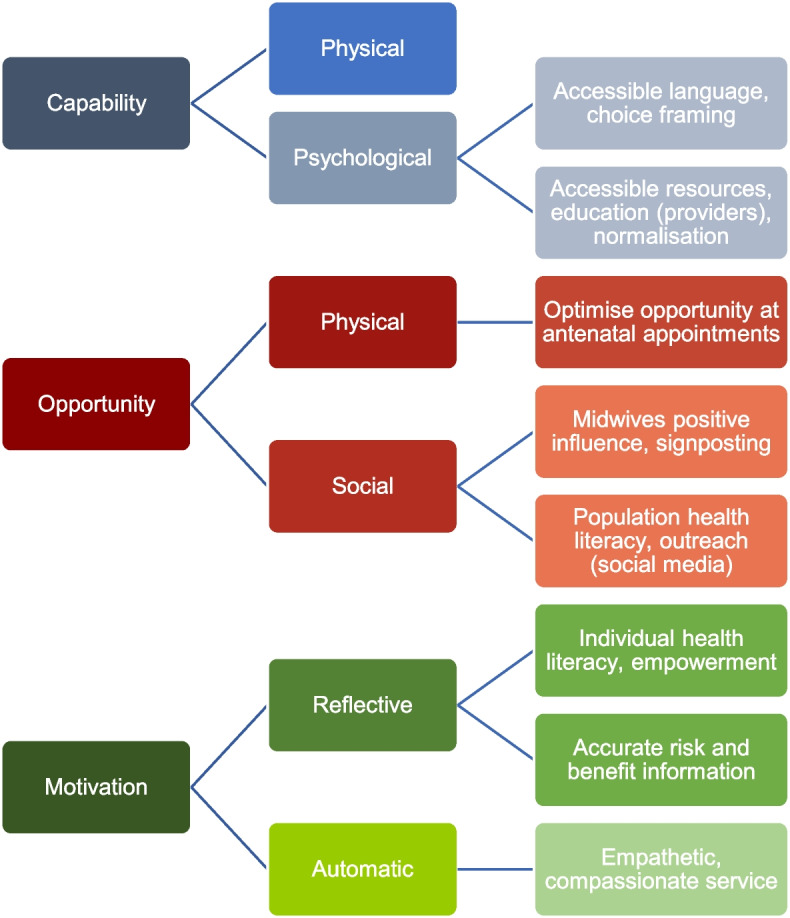
Recommendations at the patient, provider, and service level to promote informed choice and improve COVID-19 vaccine uptake among pregnant women

The COM-B model provided the thematic structure to identify behavioural determinants of COVID-19 vaccine behaviour during pregnancy (Table 3). The only COM-B domain not represented in the data was physical capability, which may be reflective of this demographic cohort and the target behaviour (i.e., the influence of personal physical skills/ability was less relevant). The results presented draw primarily from the analysis of in-depth interview data; however, where a contrast was identified in the focus group data, this has been highlighted [see Additional file 3 for interview coding tree and Additional file 4 for focus group data]. The subthemes capture positive and negative factors that have influenced COVID-19 vaccination. Certain experiences crosscut COM-B domains and are discussed within the narrative. Finally, Fig. 1 provides a schematic overview of recommendations for maternity services and providers to improve COVID-19 vaccine uptake.

### Psychological capability

#### Subtheme 1.1: Consistency and reliability of COVID-19 vaccine information and research

Most interview participants felt that in general COVID-19 vaccine information was inconsistent and all said that either the newness of the vaccine or the lack of long-term research was a factor which influenced their decision making (Table 3).

Most participants reported feeling overwhelmed by the magnitude of health information, particularly by the conflicting advice provided by healthcare professionals, which contributed to doubt and uncertainty, about what decision best supports their wellbeing. [26]

A frequently used example was the perceived “quick” change in COVID-19 vaccine recommendations during pregnancy (Table 3). Interviewees reported a lack of evidence-based information to inform pregnant individuals about what is in the vaccine, which alongside concerns about the long-term health impact, generated worry and apprehension (Table 3). Conversely, interviewees acknowledged the success of other vaccine programmes and felt reassured about their safety because of the length of time they have been available:

*…my baby has all her vaccines. I would never not get her vaccines, but I think I'm just basing it on the fact that they've been around for years, and there is evidence to suggest there are no bad side effects. It's just evidence based…* (Interviewee 5, 25–29 years).

#### Subtheme 1.2: Balancing authentic and accessible risk–benefit information and choice-framing

Most interview participants felt that information about the risks, benefits and safety of the COVID-19 vaccine was not always provided or, when provided was not always balanced. Several felt that health advice and information was positively biased and neglected the disclosure of risk information (Table 3). A few felt that vaccine risk and safety information was somewhat biased towards the benefits with aspects like side effects and the impact on babies not described. Interview participants also felt the language and terms used were not easy to understand. Subsequently, it was difficult to trust in and feel reassured by the information and advice provided. There was a sense that population health literacy around vaccines could be improved to support understanding:

*There's no education into vaccines, even at school there's nothing. I suppose if you have that continuing education, it's something you can make an informed decision* (Interviewee 2, 35–45 years).

A few interview participants suggested that positive framing of the safety data and benefits both for mother and baby (as opposed to ambiguous risk information and ‘scare tactics’) could help with making an informed decision.

*Try and keep it more positive because I think women have enough to worry about…* (Interviewee 7, 25-29 years)

A single participant stated that it would be valuable to arrange dedicated time to talk about the vaccines, as they consider it to be a major decision and therefore the allocated time to discuss with a healthcare professional should be proportionate.

Most participants felt that advice to get the vaccine should use confident and reassuring language:

*..it would influence people more to get it because it's really recommended for you, not just, 'We think you should do this.' If it's there in writing on your wee checklist, you're like, 'Right, okay, I need to get that done.* (Interviewee 1, aged 30-34 years)

Participants also stressed the importance of being empowered to make their own health decisions (Table 3).

### Social opportunity

#### Subtheme 2.1: Persuasion of personal relatedness

All interviewees felt that experiences from family, friends or colleagues had a prominent influence on vaccine-related decisions. Many also reporting turning to friends who worked in healthcare as trusted sources.

*Two of my friends work in healthcare, as nurses and one works in the pharmacy in the hospital and just hearing things that they had to say because I felt like they were well informed and they both have children as well*” (Interviewee 1, 30–34 years).

Interview participants reported that social media stories can be misleading, yet several reported that their vaccine-decisions were influenced by social media posts (Table 3). Participants’ experiences suggested that the influence of personal stories from friends or on social media have a key role in determining vaccine-decisions (in either direction), so harnessing personal stories to reassure safety concerns could be of benefit (Table 3). Within this subtheme, focus group perspectives (participants who received the vaccine) suggested that social media stories were less of an influence, and rather, these participants were influenced more by family, friends, and colleagues (see S2).

#### Subtheme 2.2: Trust in health professionals and professional organisations

Most interview participants reported trust and confidence in their midwife’s advice and trust in official public health and scientific advice. This was primarily because they felt their midwife would have their baby’s health as their main focus (Table 3). Nevertheless, for many participants, the decision to not get the vaccine was driven by the perceived lack of information or explicit advice or reassurance from their midwife or other healthcare professional.

*… Had they said, 'Yes, you should take it, these are the reasons why, this is the research,' then I probably would have* (Interviewee 2, 35–45 years).

### Physical opportunity

#### Subtheme 3.1: Vaccine delivery within familiar healthcare pathways.

Most interview participants felt that being able to access vaccines during routine health appointments would both improve accessibility but also provide reassurance because of the familiarity both setting and staff.

*…if you were leaning towards getting the vaccine, it would probably be more reassuring, if at your appointments, the midwives that you deal with, that you were able to get it after your appointment rather than having to go and book it…* (Interviewee 5, 25–29 years).

Other vaccine incentivising factors discussed included pregnancy-specific sessions, convenience, and the feeling that it was both clean and safe (Table 3).

### Reflective motivation

#### Subtheme 4.1: Unnerved by the unknown (vaccine risk and safety)

Interviewees expressed doubts and concerns about safety and the effects of the vaccine for the baby (Table 3). Concerns about the short-term and long-term risks were reflected in participant narratives. Some participants referred to the lack of long-term research as discussed previously within the ‘Psychological Capability’ domain. Others were concerned about not being able to take time out from work or family commitments if they experienced side effects (Table 3).

Several participants also reported that a barrier to uptake was that the vaccine is an unknown / unfamiliar substance, which generated fear and mistrust (again, pertaining to the discussion within the ‘Psychological Capability’ domain; Table 3). Most interview participants felt that the personal benefits of the vaccine were unclear (Table 3).

For most interviewees, the decision about vaccine uptake was collectively influenced by the perceived low personal threat of COVID-19 alongside their concerns about the side effects of the vaccine. This reflected a conscious, personal cost–benefit analysis.

*The way I was looking at it, essentially it would be fine, the baby. I reckon that I probably would be fine, compared to if I got a vaccine, it's a gamble either way. I would rather try and avoid Covid than get a vaccine, while either could impact the baby* (Interviewee 5, 25–29 years).

#### Subtheme 4.2: Confidence in personal health agency

For many participants, fears driven by concerns about the risks of adverse side-effects to their baby generated uncertainty about what decision they should make, which decreased confidence in their own health choices. Acquiring COVID-19 was considered something that participants felt they had little control over, whereas choosing to get the vaccine placed the onus on them. Irrespective of the action they took, many participants felt responsible for any harms to their baby.

*.. it made me feel like if I don't take this [the vaccine] and I get Covid and something happens to my baby, that will be my fault. But then also if I do take this vaccine, and something happens to my baby it's also my fault…* (Interviewee 8, 30–34 years).

Some of the interview participants believed that not getting the vaccine was safer for their baby and could beneficially support natural immunity in the baby from infection (Table 3). Honouring and supporting personal health choices was strongly valued. Participants expressed the desire and need to feel that they were making the healthiest and ‘best’ decisions for them and their baby. This perceived inability to make an autonomous, confident, and informed decision with respect to the vaccine appears to underpin the discomfort of the indecision experienced. Focus group participants also highlighted the importance of feeling optimistic that getting the vaccine was a healthy decision (see S2).

### Automatic motivation

#### Subtheme 5.1: Fear drives indecision

Most of the interview participants described how indecision about the vaccine was driven by fears about the unknown risks and consequences of the vaccine (Table 3). This fear was exacerbated by scary “stories” pertaining to the unknown or adverse impact of the vaccine, primarily for the baby.

*… I was scared and was thinking, 'If I get it, and something happened to my wain [baby]… I would automatically feel like, because I got that vaccine, I'm the one that injured my wain…* (Interviewee 3, 18–24 years).

#### Subtheme 5.2: Feeling unheard and cornered

Some of the interview participants felt trapped by their own conflicting thoughts about whether or not it was safe to get the vaccine, combined with a sense of pressure to receive the vaccine. There was a sense of feeling negatively judged by healthcare professionals including midwives, as well as the wider public who may be attending vaccine clinics at the same time (Table 3). Some participants felt that there was a lack of empathy, owing to the personal nature of what it feels like to be pregnant and making decisions about their own and their baby’s future health at that time.

**Table 3:** Selected interviewee responses mapped to subthemes within the COM-B framework

COM-B	Subtheme	Sample of responses
**Psychological capability**^**a**^	**Consistency and reliability of COVID-19 vaccine information and research**	*“It was the change so quick. With my first baby, it was 'No, don't get it,', like no chance, and then all of a sudden the same midwives are saying, 'You need to get it.' In a short space of time you can say it's safe” (Interviewee 5, 25 – 29 years)* *“I personally don't know what's going into a vaccine… and I don't think it's been researched enough—what could happen if a pregnant woman gets the vaccine, what it could do to her body, what it could do to her baby. It's different when you're the average person, not pregnant…” (Interviewee 6, 18 – 24 years)* *[trust in safety of other vaccines as]…”they've been around long enough, there's enough research into them” (Interviewee 8, 30 – 34 years)*
	**Balancing authentic and accessible risk–benefit information and choice-framing**	*“There's a lot of medical terminology and jargon and stuff and if you had, if it was laid out in more layman's terms, this is the research, this is the statistics, good and bad, because there's no point in hiding the bad, if there is going to be a risk, then name what the risk is… I think the more transparent you are, the more likely people are to be accepting of something…” (Interviewee 2, 35 – 45 years)* *“If they were able to say, 'All these babies have been born and they've been fine, and if you don't get it, your baby could be very, very sick,' but they couldn't back any of these things up, there was no solid information on side effects. It was just saying 'None we know of,'…” (Interviewee 5, 25 – 29 years)* *“I think in your pack, have the information and the leaflets about the injections and the thing, go, 'Look, this is what's offered to you during pregnancy, it's totally optional, what do you want to do? Go home, have a look over it, read over it, and make your own mind up and it'll tell you on your leaflet you book that with your doctors at such-and-such a date'” (Interviewee 8, 30 – 34 years)*
**Social opportunity**^**b**^	**Persuasion of personal relatedness**	*“..it was my mum, who now has, or is going last month or this month to see about having long COVID… and she's like, 'Do not get your vaccine, do not get it, I'm telling you, don't do it, especially in pregnancy'” (Interviewee 3, 18 – 24 years)* *“…maybe studies where you've interviewed women that have had babies and interviewed women that have it, then maybe just done a study on the wee ones that are born then after, their own health and their own behaviours” (Interviewee 4, 30 – 34 years)* *“I would've been a big TikTok person back then, you know, and that's where you would hear people's personal stories on what they were going through. There was a woman I think, and she had COVID. No, she wasn't pregnant or anything, but she had to learn how to walk again, she had to learn how to talk again, she got so ill after 3 days of having the vaccine and I just thought, like if that's because of the vaccine, that's weird …” (Interviewee 6, 18 – 24 years)*
	**Trust in health professionals and professional organisations**	*“…I feel like I really did trust them [midwife] as much as anybody else, because they were there to help me and help my baby throughout that. Covid or not Covid, your midwives are there to support you” (Interviewee 7, 25 – 29 years)*
**Physical opportunity**^**c**^	**Vaccine delivery within familiar healthcare pathways**	*“They had a drop-in in (town) for pregnant women so I felt even safer going to that. I was like, excellent, it's going to be all people, same situation as me, everyone's going to be cautious” (Interviewee 1, 30 – 34 years)* *“I think those mobile vaccine clinics were brilliant, really. I think they should keep them actually because they were very, very good and they should have, it shouldn't be tedious when you go to your whatever, appointment, your 20 week appointment or whatever, you're just, it's a one stop shop really” (Interviewee 2, 35 – 45 years)*
**Reflective motivation**^**d**^	**Unnerved by the unknown (vaccine risk and safety)**	*“It was probably fear of the unknown and not really for me as such, but more for my baby …” (Interviewee 2, 35 – 45 years)* *“It was a decision I didn't make lightly, and I think I changed my mind about 20 times back and forth and back and forth, but in the end, it was just that I didn't want to take a vaccine that I didn't know if it would then in a few years' time come back to say, 'Your baby has this because you took the vaccine.'..” (Interviewee 4, 30–34 years)* *“Because the baby's supposed to be getting all your antibodies and everything that they need through you, through you eating and being healthy and whatever. So, because it was so uncertain at the time, you don't know what benefits there were of you getting the vaccines, and what benefits it would have on the baby” (Interviewee 5, 25 – 29 years)* *“…people were getting the vaccine and still getting COVID so it's not as if it prevented it” (Interviewee 6, 18–24 years)* *“The only benefit that they told me was if I was to catch COVID I would have had less chance to have been hospitalised but healthy people are on a low risk anyway of being hospitalised with COVID-19 anyway” (Interviewee 8, 30 – 34 years)*
	**Confidence in personal health agency**	*“If I was pregnant and contracted a virus, my view would have been, and this may be wrong, it's nearly better because then the baby develops a particular immunity” (Interviewee 2, 35 – 45 years)* *“Once I got that last thing of COVID, I was like, 'I don't know, I maybe should have got my vaccine.' That's when the guilt sort of hit in” (Interviewee 3, 18 – 24 years)* *“I feel it's a very personal choice. I do feel it's a very personal choice, and I think you can give all the information in the world, but if it doesn't sit right, it doesn't sit right. As I say, I was given every bit of information and everybody around me, but it just didn't sit right with me” (Interviewee 4, 30 – 34 years)*
**Automatic motivation**^**e**^	**Fear drives indecision**	*“I definitely felt like they were telling me to take it and that Covid-19 wasn't going anywhere, and that really, I had a decision to make but really, I should do that quite quickly because there were pregnant women who had had it and were in ICU… And I kept saying that I didn't want to put my baby at risk” (Interviewee 7, 25 – 29 years)*
	**Feeling unheard and cornered**	*“They didn't really tell me much about the vaccine itself, to be honest. It was more, 'Have you had your vaccines?' Patronising more, you know, very patronising..” (Interviewee 3, 18 – 24 years)* *“Anybody who's not pregnant during that won't know the internal turmoil of that and to get to a decision really should have a lot of respect…” (Interviewee 7, 25 – 29 years)* *“there was one of the girls, the community midwives in the thing, my partner he had come to pick me up and I actually came out crying because she pressurised me that much, she kept going to me, 'You know it's not just you, you have to think about anymore? You have a baby inside you” (Interviewee 8, 30 – 34 years)*

^a^Participants’ knowledge and understanding of COVID-19 infection and vaccination information and guidance, the ‘framing’ of this information, and the impact of this on decisional processes

^b^The role of social influences on interview participants’ COVID-19 vaccine decisions and subsequent behaviour

^c^The influence of environmental context and resources, to include access and delivery of the vaccine

^d^Participants’ beliefs about the consequences of the COVID-19 vaccination, as well as personal capabilities and sense of optimism

^e^How emotional experiences influenced COVID-19 vaccination decisions

## Discussion

This study highlights that for pregnant women, the decision to accept a COVID-19 vaccine is complex and personal. Consistently, decisional conflict was observed for the majority of interviewees. Decisional conflict is a phenomenon that captures the oscillation between choices that generate cognitive or mental discomfort and make it difficult to decide which choice to make [26]. This phenomenon cross-cut themes, which provided greater context as to what underpinned this experience. The relative newness of the COVID-19 vaccine and the lack of long-term safety research was a prominent barrier of vaccine uptake and an important driver of decisional conflict. This is reflective of misunderstanding or lack of knowledge about the nature of vaccine research (Psychological Capability). Participant concerns were primarily about the potential long-term effects of the vaccine on the baby and not solely about the risks to themselves (Reflective Motivation). Many said that they wanted to avoid harm to their baby child, and the anticipated guilt they would feel if any subsequent health issues emerged. It is apparent that family, friends and healthcare professionals (especially midwives) all have a significant influence on women’s decision to get the COVID-19 vaccine (Social Opportunity). Participants were wary of information they read on social media, but nonetheless exposure to this information did create doubt.

Several of the barriers identified in this study reflect those reported elsewhere. For example, previous studies have reported barriers to COVID-19 vaccine uptake to include the risk of adverse effects to the developing foetus and lack of safety and efficacy data surrounding vaccination in pregnancy [27–30]. The latter has largely been driven by the exclusion of pregnant women from clinical trials, which needs to be addressed [27, 28, 31]. In addition, several studies have now demonstrated the benefits to the foetus conferred by cross-placental transfer of antibodies against SARS-CoV-2 [32–34].

It is recognised that the patient – healthcare professional (HCP) dyad is fundamental to ensuring confidence and motivation towards vaccination [35, 36]. This was evident in our study, where participants emphasised their trust in midwives and clinicians. This is consistent with a previous study in Northern Ireland which looked at barriers to pertussis and influenza vaccine uptake in pregnancy [21]. However, it has been reported that a significant proportion of healthcare professionals are vaccine-hesitant themselves [35]. This is reflected in vaccine uptake where approximately 20% remain unvaccinated, with medical and dental staff more likely to get vaccinated when compared to nursing and midwifery [37, 38]. The impact that this has on pregnant women was not investigated here, but interviewees did report sensing a degree of ambivalence when their midwife mentioned/offered them the vaccine. Specifically, midwives did not strongly endorse the vaccine, nor did they discuss the vaccine in depth. In the context of the current study it is not clear whether this was due to the personal opinion of the midwife (i.e. they did not agree that the vaccine should be offered) or was reflective of a lack of confidence in the information they possessed to confidently discuss this with their patient. This highlights the need to consider how best to support HCPs to communicate balanced risk–benefit information to their patients using accessible language and consistent sign-posting to the most-up-to-date evidenced-based information [27, 31]. Moreover, this study suggested that pregnant women would feel more emotionally supported if HCPs showed greater empathy towards their apprehensions about the vaccine and concerns about the safety and allowed the time and space to converse about this (Automatic Motivation). This also prompts the need to reduce hesitancy amongst HCPs by improving immunisation training and COVID-19 vaccine literacy [30, 35, 39]. Further research utilising the COM-B model to identify interventions to reduce vaccine hesitancy amongst healthcare professionals is warranted.

Family and friends have a key role in decision making, and for this reason it is important to improve health literacy widely as well as in pregnant women specifically [39]. This includes a basic understanding of how vaccine candidates are identified, trialled, and authorised and the influence of changing evidence as well as information about ongoing safety monitoring. Our study highlighted that often pregnant women were advised by family and friends not to get the vaccine, despite being a recognised clinical risk group for more severe COVID-19 disease. The lack of recognition, from the wider family and friends’ network, that the virus poses a greater risk than the vaccine [27] is a failure in public health messaging which could be improved with the support of personal and public involvement professionals to increase the salience and accessibility of this information [40]. It is clear there is a strong influence of perceptions, for example, perceived lack of benefit of vaccination, or perceived risks of COVID-19 or the vaccine itself. Tools such as the health belief model in particular, because of the emphasis on perceived risks and benefits, could be used to design interventions to address this [41].

Improving and simplifying access to the COVID-19 vaccine emerged as a facilitator for uptake, such as vaccine provision at routine antenatal appointments (Physical Opportunity) [31]. Getting vaccines within the familiarity of the antenatal appointment not only provides psychological safety, but normalises COVID-19 vaccine uptake and optimises convenience [31, 42]. It would also help to address the perceived difference in risk aversion reported by the participants of this study that was driven by the belief that the general population (those who are not immunocompromised) should be less concerned about potential exposure to COVID.

Finally, an interesting observation was that seven of the eight participants who chose not to get the COVID-19 vaccine while pregnant did receive the antenatal vaccines for pertussis and influenza. This is in contrast to a previous study in the UK which showed that intention to get a COVID-19 vaccination was strongly correlated with pertussis vaccine uptake, although this was prior to COVID-19 vaccines being licensed [43]. While further research is required, this highlights the challenge of vaccine hesitancy and shows that the decision-making around the COVID-19 vaccine was unique. Vaccine-specific decision-making has also been observed in Turkey, where acceptance of the tetanus vaccination (part of the antenatal programme) was higher than both influenza and COVID-19 [44]. In the UK context, this highlights a potential opportunity to normalise the COVID-19 programme alongside longstanding antenatal vaccines.

### Strengths and limitations

The strength of this study is that it focussed on the time period from which COVID-19 vaccination for pregnant women was endorsed by both JCVI and RCOG. The study was also rolled out quickly to gather knowledge that could be used to shape the autumn COVID-19 vaccine booster programme among pregnant women, during 2022. However, given the tight timescales, and limited funding, the number of participants is small. Despite this, it was reassuring to see that data coded for the focus group corroborated the codes and subthemes extracted from the interview data. The participants were chosen to ensure representation across Northern Ireland, younger and older women of childbearing age, and by socioeconomic status. However, one gap in our recruitment strategy was representation from minority ethnic groups as a result of the small sample size, tight recruitment timescales and smaller eligible cohort in the Northern Ireland population, given the inclusion criteria for this study. This is a significant limitation given what is known about the risk of severe COVID-19 disease among minority ethnic groups who are also less likely to accept the COVID-19 vaccine [43, 45, 46]. It is conceivable that the barriers and facilitators for minority ethnic groups will differ and therefore further work is needed to develop targeted interventions for these populations.

## Conclusion

Our study highlighted the various psychological, social, and environmental factors underpinning the decisional conflict a pregnant woman faces when considering COVID-19 vaccination. In this regard, focussing on risks and benefits to mothers alone will likely have limited influence. It is important that pregnant women can access balanced risk and benefit information, for them and their child, framed using language and terms that are easy to understand and that positively emphasise the benefits that are most relevant to pregnant women. Healthcare professionals involved in the antenatal care pathway should also feel informed and confident in their capacity to discuss the risks and benefits with pregnant women. Moreover, they should feel empowered to communicate in a way that is empathetic and accommodates the difficult emotions experienced by their patients. The key insights drawn from this research provide a useful starting point to inspire the co-design of patient and public involvement supported public health interventions that focus on improving the delivery of vaccine information and communication and empowering HCPs to support pregnant women in their decision making. Further, interpreting the findings through the lens of the COM-B framework enables public health researchers and professionals to consider the relevant intervention functions using the Behaviour Change Wheel. These findings should be corroborated through further longitudinal empirical research to support reliability and generalisability.

### Supplementary Information

**Additional file 1.** Recruitment screening questionnaire.

**Additional file 2.** Discussion guide used to facilitate semi-structured interviews and the focus group.

**Additional file 3.** Coding tree for semi-structured interviews.

**Additional file 4.** Coding tree for focus group.

## Data Availability

The datasets used and/or analysed during the current study are available from the corresponding author on reasonable request.
