# Supplementary material for: Using the COM-B framework to elucidate facilitators and barriers to COVID-19 vaccine uptake in pregnant women: a qualitative study
Source: BMC Pregnancy Childbirth. 2023 Sep 6;23:640. doi: 10.1186/s12884-023-05958-y (PMC10481472; doi:10.1186/s12884-023-05958-y)
Supplement: Supplementary file 1 — Additional file 1. Recruitment screening questionnaire. [file 12884_2023_5958_MOESM1_ESM.pdf]

22-018180 PHA COVID-19 vaccine during pregnancy research Recruitment Screener

INTRODUCTION:

Hello, my name is [Recruiter]. I am currently arranging a research project and would like to ask you some questions and, if you are eligible, invite you to participate.

I need to let you know some information before we go any further. I am working on behalf of Ipsos, an independent research agency. It has been commissioned by the Public Health Agency (PHA) to organise some research on their behalf. This research will help the PHA in Northern Ireland to explore views of new and expectant mothers on uptake of the COVID-19 vaccine.

The answers that you give to me to today will only be shared with Ipsos. These answers will be used to establish whether you are eligible to take part in the study. This exercise is purely a research project, so anything you say during the research exercise itself or during this interview will remain confidential.

You have the right to withdraw your consent to process the information you provide or object to our processing of your information. You can let me know at any time during this conversation if you would like to do this. Should you decide to withdraw your consent after this conversation, you can call me on [insert telephone number] to let me know.

The research activity and this interview will be conducted in accordance with the Market Research Society Code of Conduct, and the information you provide will be treated in accordance with GDPR and data protection law. Our privacy notice for this research is available to you, please let me know if you would like a copy. For this project, Ipsos will securely remove your personal data from their systems within six months of project close. Personal data will be retained for this long for quality control purposes.

As I mentioned, this interview is just to establish eligibility for the research project and will take around 10 minutes. The research project itself will involve attending a 1.5 online focus group on [insert relevant group time and date] or attending a 45 minute virtual interview. The online group or interview will take the form of a video focus group, which will be conducted on Microsoft Teams. You will need to have access to a device with a webcam and microphone and have a strong internet connection.

Eligible participants will receive <insert appropriate incentive amount> for their time.

Q1.	**Do you consent to take part in the research on this basis?**<br>SINGLE CODE ONLY		
	Yes	1	CONTINUE
	No	2	THANK AND CLOSE

Q.2 Would you be available to attend an online focus group on ____________________________?

Yes	1	CONTINUE
No	2	THANK AND CLOSE

READ OUT

Q.3 Do you or any of your family, or close friends work or have ever worked, OR are currently or have ever been involved in any of the following ……….?

SINGLE CODE ONLY

Advertising	1	SCREEN OUT
Marketing/Market Research	2	
Public Relations (PR)	3	
Journalism/ the media	4	
The Public Health Agency	5	
COVID-19 vaccine roll out	6	
None of these	00	CONTINUE

Q.4 Have you participated in a group discussion, workshop or large-scale market research event in the last 12 months?

SINGLE CODE ONLY

Yes	1	SCREEN OUT
No	2	CONTINUE

Q.5 Which of the following describes how you think of yourself?

SINGLE CODE ONLY

Male	1	SCREEN OUT
Female	2	CONTINUE
In another way	3	SCREEN OUT

Q.6 **What was your age on your last birthday?**
STATE EXACT AND CODE

Exact age: ___________	18-34	1	RECRUIT AS PER RECRUITING INSTRUCTIONS
	35-49	2	
	50+	3	SCREEN OUT
	DK/REF	00	

Q.7 Are you currently pregnant or have you been pregnant since April 2021?
SINGLE CODE ONLY

Yes, currently pregnant	1	CONTINUE
Yes, delivered my baby since April 2021	2	
No	3	Screen out

ASK IF DELIVERED A BABY SINCE APRIL 2021 (CODE 1 AT Q7)

Q.8a What month was your baby born?

Record month<br><br>__________	April-June 2021	1	Recruit a spread across vaccine roll out period
	July - September 2021	2	
	October-December 2021	3	
	January - March 2022	4	
	Dk/REF	00	Screen out

ASK IF CURRENTLY PREGNANT (CODE 1 AT Q7)

Q.8b What is your current trimester of pregnancy?

1st trimester	1	CONTINUE
2nd trimester	2	
3rd trimester	3	

ASK IF DELIVERED A BABY SINCE APRIL 2021 OR CURRENTLY PREGNANT (CODES 1 OR 2 AT Q7)

Q.9 Is/was this your first pregnancy?

Yes	1	**CONTINUE**
No	2	

ASK ALL

Q.10 How many doses of the COVID-19 vaccine have you had during your pregnancy when you knew you were pregnant?
SINGLE CODE ONLY
PROBES TO PRECODES

I received one dose of the COVID-19 vaccine when I knew I was pregnant	1	CONTINUE
I received two doses of the COVID-19 vaccine when I knew I was pregnant	2	
I received three doses of the COVID-19 vaccine when I knew I was pregnant	3	
I haven't taken any doses of the COVID-19 vaccine while I've known I'm pregnant	4	
I took one dose of the COVID-19 vaccine but did not know I was pregnant	5	CLOSE AND RS
Refused	6	

ASK IF PARTICIPANT HAS NOT TAKEN ANY DOSES OF THE COVID-19 VACCINE (CODE 4 AT Q10)

Q.11 How likely or unlikely are you to consider vaccination during this pregnancy?

Very likely	1	CONTINUE AND RECRUIT TO GROUP OR INTERVIEW AS APPROPRIATE
Fairly Likely	2	
Indifferent	3	
Fairly Unlikely	4	
Very unlikely	5	
Don't know	98	CLOSE AND RS
Prefer not to say	99	

Q.12 Which of the following areas in Northern Ireland do you live in?
SINGLE CODE

Please specify postcode:

____

Belfast Health and Social Care Trust	1	RECRUIT A SPREAD
Northern Health and Social Care Trust	2	
South Eastern Health and Social Care Trust	3	
Southern Health and Social Care Trust	4	
Western Health and Social Care Trust	5	
Prefer not to say	99	THANK AND CLOSE

Q.13 Which ethnic group to you belong to?

White	1	CONTINUE AND RECRUIT TO GROUP OR INTERVIEW AS APPROPRIATE
Asian/Asian British	2	
Black/African/Caribbean/ Black British	3	
Mixed/multiple ethnic group	4	
Other (please specify)	5	
Don't know	98	CLOSE AND RS
Prefer not to say	99	

Q.14a.	**What is your current employment status?**<br>SINGLE CODE ONLY		
	In full-time employment	1	CONTINUE
	In part-time employment	2	
	Currently not in paid employment	3	
	In full-time education/studying	4	
	Look after the home / children	5	
	Carer	6	
	Retired	7	
	Prefer not to say	8	SCREEN OUT

Q.14b	**And could you tell me what it is you do for a living?**		
	Position/rank/grade		RECORD FOR INFORMATION
	Industry/type of company		
	Prefer not to say		SCREEN OUT

Q.14c	**And could you tell me what the chief income earner in your household does for a living (if not yourself)?**		
	Position/rank/grade		
	Industry/type of company		
	Number in charge of		

READ OUT

Q. 14d Please tell me your estimation of the household's annual income before tax. If you prefer tell me the letter beside the income band.			
G Under £5,000	01	N £35,000-£39,999	08
H £5,000-£9,999	02	O £40,000-£49,999	09
I £10,000-£14,999	03	P £50,000+	10
J £15,000-£19,999	04	Not applicable	11
K £20,000-£24,999	05	Refused	12
L £25,000-£29,999	06	DK	00
M £30,000-£34,999	07		
Social Grade			
Prefer not to say		SCREEN OUT	

Q.14 Record occupation here
STATE EXACT AND CODE

____

____

A	1	RECRUIT A SPREAD
B	2	
C1	3	
C2	4	
D	5	

E	6

Q15	**The research is a group discussion/interview to be conducted online with a researcher. You will need access to a computer/laptop/tablet that has a webcam and microphone enabled. Detailed instructions will be sent to you about this when you are sent the link to join the session. Members of the research team will also be on hand to assist if necessary.**<br><br>**Are you happy and able to participate on this basis?**		
	Yes	1	CONTINUE
	No	2	THANK AND CLOSE

Q16	**It may be necessary for Ipsos to contact you by email or telephone after the research has taken place to follow up on ideas generated during the discussion. You would only be contacted if strictly necessary and only in connection with this research. Are you happy to agree to be re-contacted on this basis?**		
	Yes	1	CONTINUE
	No	2	REFER TO OFFICE

Recruiter Note:
This question is to ensure that participants can be contacted directly in relation to this research. It does not permit re-contact of participants for any other purpose or panel building.

Interviewer name (CAPS):.................................

I confirm that I have conducted this interview over the telephone/ face to face with the named person of the address attached and that I asked all the relevant questions fully and recorded the answers in conformance with the survey specification and within the MRS Code of Conduct and the Data Protection Act 1998.

Interviewer Signature:.....................................

Date:...

Participant's DETAILS (PLEASE COMPLETE IN BLOCK CAPITALS)

Title: Mr/ Mrs/ Miss/ Ms/ Other: ______________________________

First name: ______________________________

Surname: ______________________________

EMAIL ADDRESS: ☐☐☐☐☐☐☐☐☐☐☐☐☐☐☐☐☐☐☐☐☐☐ ☐☐☐☐☐☐☐☐☐☐☐☐☐☐☐☐☐☐☐☐☐☐☐☐☐☐☐

Mobile Number: ☐☐☐☐☐☐☐☐☐☐☐

House Number/Name: ______________________________

Street: ______________________________

____

Town/City: ______________________________

Postcode: ☐☐☐☐ ☐☐☐

Telephone Number

☐☐☐☐☐☐☐☐☐☐☐

Bank details (for incentive payment)

Account number______________________________

Sort code ______________________________

CHECKLIST: PLEASE ENSURE YOU HAVE DONE ALL OF THE FOLLOWING BEFORE CLOSING

Have you?
(please circle once completed)

Re-confirmed time and date of group?	1
Given details on platform/online group?	2
Reconfirmed incentive amount and bank details?	3
Recorded mobile number & email address?	4

INTERVIEWERS DECLARATION

This interview was conducted in accordance with the Market Research Code of Conduct. The respondent is not a relative or friend of mine.

Face to Face	1
Telephone	2
Street	3
Door to Door	4

Interviewer's name:

____

Interviewer's signature:

____

Date: ______________________2021
