# Supplementary material for: Using the COM-B framework to elucidate facilitators and barriers to COVID-19 vaccine uptake in pregnant women: a qualitative study
Source: BMC Pregnancy Childbirth. 2023 Sep 6;23:640. doi: 10.1186/s12884-023-05958-y (PMC10481472; doi:10.1186/s12884-023-05958-y)
Supplement: Supplementary file 2 — Additional file 2. Discussion guide used to facilitate semi-structured interviews and the focus group. [file 12884_2023_5958_MOESM2_ESM.pdf]

Market research company discussion guide

Introduction

- Introduce self/ management research company – stress role as an independent research organisation and that we are here to listen to opinions.
- Outline background and objectives of the project
- Explanation of discussion 'rules'
- Explain participant's rights under GDPR
- Mention recording/viewing/filming of proceedings and obtain permission to record/film. Reinforce anonymity/confidentiality
- If applicable, explain note takers/client presence
- Housekeeping – tech issues etc
- Participant introductions: ask for name, age, and give a little bit of information about themselves

Warm-up (it is important to ask all participants this to warm them up)

Can you tell me about your current or recent pregnancy and how it's been going for you in the context of the pandemic?

I'd like you think a little about vaccinations and particularly taking vaccines during pregnancy.

I'm going to read out the start of two sentences and I would like you to finish them based on how you personally feel.

Vaccinations during pregnancy are…

The thought of taking the COVID-19 vaccine during pregnancy makes me feel …

Knowledge and understanding of COVID-19

What, if anything, do you know about COVID-19 (also known as coronavirus)? PROBE FULLY

- Transmission
- Symptoms (fever/temperature; new, persistent cough; loss of smell or taste)

During your pregnancy, have you changed your behaviour as a result of the COVID-19 pandemic at all?

In what ways?

PROBE FULLY WITH:

- Self-isolation;
- Social distancing;
- Reduced contact with friends/family; and,
- Stopped visiting places I would normally visit.

What impact, if any, do you think COVID-19 can have for pregnant women and their baby? PROBE FULLY

- How might this affect you? Probe higher risk of being hospitalised, admission to ICU
- How might this affect your baby? Probe increased risk of pre-eclampsia, preterm birth and stillbirth for their baby

COVID-19 vaccination during pregnancy

What sources, if any, have you used to get information about the vaccine, COVID and pregnancy?

- Why did you use this source/these sources for information?

Who would you say you trust most to give you information about your health in pregnancy? PROBE FULLY

- Why would you most trust them?

During your pregnancy, have healthcare professionals (e.g. midwives) spoken to you about COVID-19 vaccination?

- Who specifically spoke to you about the COVID-19 vaccination?
- What did they tell you about it?
- Did they cover risks and benefits?
- Did the tell you how and where you could get it?
- Did they provide you with leaflets, signpost you to website, anything else?
- How did the conversation with the healthcare professional make you feel?

What, if any, questions did you have about C19 vaccination in pregnancy?

- Were your questions answered?
- Why/why not?
- What else would you have liked to know at that point in time?

What factors did you consider when you were making a decision about whether you should have the COVID-19 vaccine? PROBE WITH:

- Any side effects from a dose before pregnancy; and,
- Perceived side effects or impacts during pregnancy.

Which, if any sources, influenced your decision about whether you should have the COVID-19 vaccine? PROBE FULLY WITH:

- Views of family members;
- Views of friends;
- Own research (what specifically did you look at?);
- News reports (anything specifically?);

- Social media (which platforms); and,
- Word of mouth.

What, if any, benefits do you think would result from getting the COVID-19 vaccine?

- Any benefits for yourself?
- Any benefits for your baby?
- What about before or after your pregnancy?

What, if any, concerns have you had about getting the COVID-19 vaccine during pregnancy? PROBE FULLY TO UNDERSTAND EXACT SOURCE OF CONCERNS

- For yourself/for your baby;
- Before/after pregnancy; and,
- Side effects.

Tell me a little of how you feel about vaccinations in general? IMPORTANT TO UNDERSTAND IF VIEWS ARE AGAINST VACCINATION IN GENERAL OR JUST COVID-19 VACCINATION

- Did you also receive the influenza and pertussis vaccinations during this pregnancy?

SECTION FOR VACCINATED WOMEN ONLY

When did you receive the COVID-19 vaccine?

- Before pregnancy
  - Was this a deliberate choice?
  - How many doses did you receive? One, two, booster?
- During pregnancy
  - How many doses did you receive? One, two, booster?
  - Which trimester?

Why did you decide to get the COVID-19 vaccine? PROBE WITH:

- Felt it was the right thing to do;
- My own research made me comfortable to have it;
- I knew someone with COVID-19;
- I knew someone else who is pregnant who took the vaccine;
- New data/evidence.

What was your experience of getting the COVID-19 vaccine? Thinking specifically about:

- Accessibility – how easy or difficult was it to arrange and get to venue?
- Setting – how was the setting itself?
- Did you experience any symptoms?

- Who provided the vaccine? Moderna/Pfizer
- Feelings about safety?

SECTION FOR UNVACCINATED WOMEN

Why did you decide not to get the COVID-19 vaccine?

- Were there any other reasons why you were unable to take the COVID-19 vaccine? PROBE WITH:
- Accessibility;
- Cancelled appointments;
- Illness;
- Time off work;
- Childcare; and,
- Difficulties in getting appointments.

In what ways, if any, do you think your decision would be different if you weren't pregnant? PROBE FULLY

Do you intend to get the COVID-19 vaccine in the future?

- Why?
- Why not?

Is there anything that you feel would have encouraged you to take the COVID-19 vaccination? PROBE WITH:

- Additional information;
- Healthcare professional recommendation; and,
- More data on safety of the vaccine.

What, if anything, do you think could/should be done to make it easier for pregnant women to be vaccinated?

- Where should vaccines take place?
- Who should provide them?

Is there any information you feel should be provided to pregnant women to help them make decisions about taking the COVID-19 vaccine?

Do you think public authorities and universities should analyse individual's personal health/medical data to understand and address safety, effectiveness and inequalities in vaccine access and uptake?

Do you have any final thoughts/comments you would like to make about taking the COVID-19 vaccine in pregnancy?
