# Supplementary material for: Using the COM-B framework to elucidate facilitators and barriers to COVID-19 vaccine uptake in pregnant women: a qualitative study
Source: BMC Pregnancy Childbirth. 2023 Sep 6;23:640. doi: 10.1186/s12884-023-05958-y (PMC10481472; doi:10.1186/s12884-023-05958-y)
Supplement: Supplementary file 3 — Additional file 3. Coding tree for semi-structured interviews. [file 12884_2023_5958_MOESM3_ESM.pdf]

Supplementary Table 1. Coding Tree for interviews with participants who did not receive the vaccine, exploring the behavioural determinants of COVID-19 vaccine uptake during pregnancy

COM-B conceptual themes	Subthemes	Sample codes	Sample quotes
Psychological Capability	(1.1) Consistency and reliability of COVID-19 vaccine information and research	- Lack of research/long-term research	"Vaccinations, they are uncertain. But I suppose the more you know about them, the better. And then the longer time that you've had to research it, how many years it's been out, and you can say-, even whenever you get the flu vaccine, they give you this pamphlet of A, B, C and D and what it does and what's in it. You'd need that for the COVID one, to say, 'Oh right, okay. And there is the statistics… Probably in the distant future. Maybe with 5 or 10 years more experience of statistics and resources and whatever. But not any time soon" (Interviewee 6, 20-24 years)
		- Conflicting/changing advice	"There were a lot of things, so many mixed messages and I suppose adamant not to get it. Had they said from the outset, had they said, look, we don't know enough about it, but they weren't, they were saying it's just pregnant, women who are pregnant shouldn't be getting it. Had they have come out and said that we're doing more research onto it, or had they been more reassuring from the outset, there probably wouldn't have been an issue. It was that lack of clarity, those mixed messages and even, there wasn't even just local government, it was the World Health Organisation as well, they had oversight over a lot of the research that was going on and even at that stage they were saying it's not something they were considering. It's those reassurance from medical professionals, but it probably would be the only thing that I would have maybe considered getting it at that stage" (interviewee 2, 35-39 years)
	(1.2) Balancing authentic and accessible risk-benefit information and choice-framing	- Accessibility and availability of risk information/language	"I think it just needs to be more relatable. It needs to be more, it just needs to be more accessible. It needs to be easier for the schools, there's a lot of medical terminology and jargon and stuff and if you had, if it was laid out in more layman's terms, this is the research, this is the statistics, good and bad, because there's no point in hiding the bad, if there is going to be a risk, then

		- Positive framing of safety and benefits	name what the risk is, say what it is. I think the more transparent you are, the more likely people are to be accepting of something, do you know what I mean" (interviewee 2, 35-39 years)<br><br>"I don't think scare mongering's a good thing. I think really just giving the facts, what the facts are at the minute, just put them to women and say, 'Such and such have had the pregnancy while pregnant, the babies have been okay.' I think it's just a reassurance that the babies are okay, I think for a lot of pregnant women they don't want to get this thinking they're going to harm their child. So, I wouldn't put down on the sheet, 'So many babies have survived or so many babies have died,' even just to put across that less numbers are in ICU, less women are being hospitalised, more symptoms are mild from getting the vaccine. Just basic positive facts. Try and keep it more positive because I think women have enough to worry about than worrying, 'Oh, if I don't to take this, I'm going to die or my baby's going to die.' Even though it is a very serious topic, try and keep it as straightforward and to the point as possible." (Interviewee 7, 25-29 years)
Social Opportunity	(2.1) Persuasion of personal relatedness<br><br>(2.2) Trust in health professionals and professional organisations	- Influenced by personal stories on social media e.g. TikTok, Facebook<br>- Real stories are valuable/important sources of information<br><br>- Trust in local HCP/midwife in advising health choices<br>- Confidence in routine vaccines by local HCP	"But I just don't know with the uncertainty of what that would do with me. So, when you're listening to the new, obviously they're always prompting you to try and take it, to try and prevent this. The doctors would always be trying to tell you to do it, to take it. But I think it really matters on people listening, like I say, on TikTok, on Facebook, on Snapchat about people's personal experiences. So, then you're made more aware of it. And you only the good stuff about the news, about taking the vaccine. They never put up and say about these girls that have been-, these people that have been hindered so badly because of the vaccine. You don't hear that side, unless you're looking at social media." (Interviewee 6, 20-24 years)<br><br>"100%. Yes, I think as well because you can ask questions in a one-to-one, you're already there. I felt very confident speaking to my midwife that I was getting every bit of information that I needed, so if I had questions about that, I know they would have been able to answer them, if you were hesitant at all. I just feel like you get this list and it's, 'At this many weeks, get this-, I feel like if

			it was recommended at a certain weeks in your pregnancy, the way the flu vaccine's recommended-, is it after 18 weeks or something, I think? If that was there, it would influence people more to get it because it's really recommended for you, not just, 'We think you should do this.' If it's there in writing on your wee checklist, you're like, 'Right, okay, I need to get that done.'" (Interviewee 1, 30-34 years)
Physical Opportunity	(3.1) Vaccine delivery within familiar healthcare pathways	- Time vaccine alongside routine check-up	"I suppose your local GP or your midwife appointment. Or if you're a high-risk pregnancy and you get a consultant, then obviously them as well. But I don't think there's a specific place of where. I suppose it should just be made available at your appointments, if you wanted it say, 'Okay, the next time you come, I'll have your COVID vaccine here for you.' Maybe" (Interviewee 6, 20-24 years)<br><br>"I think if you could get that at your midwife appointment, it would be amazing, especially the fact that you've made the trip out of the house already. I know now all that's eased and stuff, the social distancing and stuff, but still if I was able to just-, went to say my 20-week appointment and got my COVID vaccine there and then, that would have been great, it would have just been so convenient. You wouldn't have felt a massive pressure to go, to take another day off work to go and stand in a queue in at a walk-in or be spending hours ringing around trying to get someone to do it. You could just get it done there and then. Even whenever you're getting say your whooping cough or your flu vaccine, if you were able to get it the same way you could have got those." (Interviewee 1, 30-34 years)
Reflective Motivation	(4.1) Unnerved by the unknown (vaccine risk and safety)	- Fear of unknown impact & unknown substance	"Like I say, if they were able to say, 'All these babies have been born and they've been fine, and if you don't get it, your baby could be very, very sick,' but they couldn't back any of these things up, there was no solid information on side effects. It was just saying 'None we know of,' and I'm just like, it wasn't reassuring enough for me, I feel like there was not enough there" (Interviewee 5, 25-29 years).<br><br>"It was probably fear of the unknown and not really for me as such, but more for my baby and just that there was a real lack of-, as I said before, there was

			no clarity, there was no evidence, there was no research, even if it had been available, to say this is safe. I remember a few news pieces, there was a poor woman who was 36 weeks pregnant, and she was rushed into hospital with COVID, I think it was out in [name of hospital] and she passed away, but they managed to save her baby. I don't know, it was just fear, it was more fear for my baby than for me. But it was the fear that I wouldn't be able to, I suppose, keep my baby alive if something was to happen to me going into hospital, do you know what I mean?" (Interviewee 2, 35-39 years)
		- Real/personal benefits during pregnancy unclear	"Again, you don't know. Because the baby's supposed to be getting all your antibodies and everything that they need through you, through you eating and being healthy and whatever. So, because it was so uncertain at the time, you don't know what benefits there were of you getting the vaccines, and what benefits it would have on the baby. It was just so early on for me, that you didn't know." (Interviewee 6, 20-24 years)<br><br>"The only benefit that they told me was if I was to catch COVID I would have had less chance to have been hospitalised but healthy people are on a low risk anyway of being hospitalised with COVID-19 anyway" (Interviewee 8, 30-34 years)
	(4.2) Confidence in personal health agency	- Belief it is a personal decision (informed choice is important)	"I think with the likes of the COVID one and with the flu or the Whooping one and all, see if they put it all in a package and say, 'Look this is the thing, it's your option, you go home and read about it' and give you a booklet and stuff so you're not with someone and they may have put on the spot at that time and place and make them feel-, so let the person bring it home, make their own choice up and not being made to feel bad or they're doing wrong for whatever choice they do make" (Interviewee 8, 30-34 years)
		- No vaccine 'safer' choice to protect baby (uncertainty)	"If something happens to my wain and they are fully vaccinated, it sounds horrible, but I can blame the doctor whereas if they were to get something and it was me personally who made that decision that no, I'm not vaccinating my wain, and they got something, I would feel like that would be 100% my fault.

			Whereas whenever it's on me, well, I'm right or wrong no matter what I do, do you know, for my body.' I think that's why I never got it. But for my wains, I don't know, whatever felt right in my head. Whatever would make me sleep at night. Oh, that sounds horrible" (Interviewee 3, 20-24 years).<br><br>"It was a decision I didn't make lightly, and I think I changed my mind about 20 times back and forth and back and forth, but in the end, it was just that I didn't want to take a vaccine that I didn't know if it would then in a few years' time come back to say, 'Your baby has this because you took the vaccine.' It was just too new for me. I took my whooping cough, all the vaccines, the flu vaccine, everything, while I was pregnant, but this one was just too new for me and that weighed it up. As I say, I went back and forth and back and forth, so I just took the decision to isolate." (Interviewee 4, 30-34 years)
Automatic Motivation	(5.1) Fear drives indecision<br><br><br><br><br><br><br><br>(5.2) Feeling unheard and cornered	- Worry and fear (scary stories and unknown impact)<br>- Unease and uncertainty (risks and unknown impact)<br><br><br><br><br><br>- Feeling trapped, pressurised and/or judged by HCP/midwife	"It was probably fear of the unknown and not really for me as such, but more for my baby and just that there was a real lack of-, as I said before, there was no clarity, there was no evidence, there was no research, even if it had been available, to say this is safe… I don't know, it was just fear, it was more fear for my baby than for me. But it was the fear that I wouldn't be able to, I suppose, keep my baby alive if something was to happen to me going into hospital, do you know what I mean?" (Interviewee 2, 35-39 years)<br><br>"It was whenever I went to the Health Centre, it was them that would've tried to get me to take the vaccine and every time I went I was always handed out the leaflets. And you do know now you're at the stage of your pregnancy if you don't take the vaccine that your baby can end up in ICU if you get it and all this, so it sort of made you feel like you were being a bad mummy, you know if caught COVID and something happened to my baby because I didn't take the vaccine. But then again, what if I did take the vaccine and something happened to my baby. Would I be a bad mummy for taking a vaccine that hasn't been out that long and there's no history behind it, so for me it felt like you were very pressurised into doing it. And I don't think any girl or any woman should be made to feel like that in that position because it was sort of a decision, no matter what you chose, it was wrong. Do you know? So, if I had have chosen,

		- Lack of empathy/not feeling listened to (by HCP/midwife)	'Yes' and something happened, I was wrong for taking that. If I had have not taken the vaccine and got COVID and something happened, I was wrong for not taking the vaccine. So, it put you in a position where you just constantly felt guilty" (Interviewee 8, 30-34 years)<br><br>"I think your emotions are high going to those appointments anyway, and especially earlier I said there was nobody there to go with me for support. So, it felt like you were going to these on your own and then being told that information, coming out on your own and then trying to digest it, feeling guilty, alone…. And then, at the next appointment she went back to the sheet and she said, 'Now, I know you don't have it, do you want to discuss this?' And I said, 'Look, I'm not going to get it.' And I think by the last appointment she just said, 'Are you getting it or not?' It was the same woman each time" (Interviewee 7, 25-29 years).
