# Supplementary material for: Using the COM-B framework to elucidate facilitators and barriers to COVID-19 vaccine uptake in pregnant women: a qualitative study
Source: BMC Pregnancy Childbirth. 2023 Sep 6;23:640. doi: 10.1186/s12884-023-05958-y (PMC10481472; doi:10.1186/s12884-023-05958-y)
Supplement: Supplementary file 4 — Additional file 4. Coding tree for focus group. [file 12884_2023_5958_MOESM4_ESM.pdf]

Supplementary Table 2. Coding Tree for focus groups with participants who received the vaccine, exploring the behavioural determinants of COVID-19 vaccine uptake during pregnancy

COM-B conceptual themes	Subthemes	Sample codes (focus groups)	Sample quotes (focus groups)
Psychological Capability	(1.1) Consistency and reliability of COVID-19 vaccine information and research	- Lack of research/long-term research	"But I was anxious up until the baby was born, I'm not going to tell you lies, and not because I disrespected the views of my doctors or I'm an anti-vaxxer, but for the reason of, there just hasn't been enough time passed, girls . The research has been done but for me, I was so scared that enough time hadn't been passed to see what the outcome of this could be." (Focus group participant 5)
		- Conflicting/changing advice	"Yes. I got my first one, actually, whenever I was pregnant and didn't know. And then, the second one, I hmm'ed and haa-ed with it because the information was changing all the time about whether a pregnant woman should or shouldn't get it, so I just didn't really know what to do. No matter how many people you asked, they were like, 'Oh, it's up to you,' more or less. It was just that there was no-, at that time, it was like, 'I don't know whether you should get it or not.' And then, I think, after I got it, it was like, 'Yes, all pregnant women should get it.' So, it was just like they were making up their mind, whether pregnant people should get it or not, so it was a bit confusing." (Focus group participant 1)
	(1.2) Balancing authentic and accessible risk-benefit information and choice-framing	- No discussion of benefits vs risks of vaccine	"No. Like not about what was in the vaccine, they literally just asked, 'Have you got it?' I think one gave me like the COVID-19 helpline thing for pregnant women, so that you could phone up, it was a midwife that was giving out the vaccinations, if you wanted more advice from that. But I never phoned it. But they didn't actually talk about the benefits and the

		- Accessibility and availability of risk information/language	risks and stuff like that, they just asked did you get it, and once you said, ‘yes’ they were like ‘that’s ok’, move on.” (Focus group participant 1)<br><br>“if you asked the question, you were basically told, 'We're not told to given anybody information about it. You have to make up your own decision on that.' They didn't have any leaflets, they didn't have anything at the time. And I was really frustrated because I understood that they couldn't tell me, but even after they knew some things, they were reluctant to talk about it. It was almost as if they were worried that if anything did go wrong that you'd come back to them and say, 'Well you said this. ' But I did my own looking around on social media, and I started following a couple of really reputable people even on social media. But you have to be selective. There was an immunologist in London, who specialises in pregnancy and he was really really good, and I was able to signpost other pregnant people to the same person who was bringing out really up to date . She was looking at all the research and letting everyone know like what's being said, and how to actually look at it from a layman's point of view, so you didn't have to have any special medical knowledge to understand what she was saying.” (Focus group participant 4)
Social Opportunity	(2.1) Persuasion of personal relatedness	- Influenced by personal stories (friends, family)**	“We had friends that were nurses at the time, and they were in the COVID wards. They were just telling us that there was say, 8 out of 10 people that was in that, and maybe 6 of them that were pregnant mums. So, I was like, 'I should probably get the vaccine then.’” (Focus group participant 1)

	(2.2) Trust in health professionals and professional organisations	- Trust in local HCP/midwife in advising health choices	"I was seeing too much on the internet, and I wasn't searching for it. You're going onto Facebook, and this is when it was really, really rife at the time, and it was all new, and everybody was putting their 2D into it. I just kept out of everything, wouldn't read it, didn't want to know. Because I was at the hospital every 2 weeks anyway when I was pregnant. And I just asked the doctor like, 'Look, I've got my appointment now for my second jab, will I go?' And he just said, 'Yes.' So, I did, and that was it." (Focus group participant 1)<br><br>"The doctor just was like, 'Well my wife's pregnant and I have done my research and I have sent her on to get her vaccines.' So, I just says, 'Well that's alright, if you're making your wife get it I'll just get it too.' Because he was an obstetric endocrinologist, like he knew what he was doing" (Focus group participant 1)
		- Confidence in routine vaccines by local HCP	And then whenever I went to my midwife appointments, they'd give me leaflets and stuff. In where I went in the area, I think at the start they would've said, 'No, we are advised to tell you to get it. You have to me that call.' Because they had the fear of maybe telling you and something happens. But I think one of their midwives got sick, so then they were all very pro-vaccine. So, they were all, 'Did you get it? Good that you got it.' So, whenever they said, 'Good that you got it.' That kind of put you a bit more at ease. Because they're the professionals. Because if you didn't, or for me, if I listened to everybody else my head would've been away with it, because people just. (Focus group participant 5)
Physical Opportunity	(3.1) Vaccine delivery within familiar healthcare pathways	- Time vaccine alongside routine check-up	"Yes, just whenever my appointments were. I asked around the time like, 'My appointments coming up, should I go to it?' And they were like, 'It's up to you.' And then I'm glad I did because every appointment after it they were like, 'What vaccines have you got? Have you got your 2 COVID jabs?'

			So, after I got it, it was advised then that all pregnant women go and got it, and they kept banging on about it, so I'm glad I did. I probably would've been sick of listening to them." (Focus group participant 1)<br><br>"Yes, it did. But I thought because they didn't feel alone, I think I would've really found that really beneficial if that had happened for me or if an environment, like at your antenatal-, or even people who haven't taken it in their post-partum period being offered it before they leave hospital . Might be a really good time as well. I know that in England, they did some things like that, where the obstetricians were offering a vaccine clinic as you were leaving the hospital because you're still at risk, high risk, 6 weeks post-partum too." (Focus group participant 4)<br><br>"It might be an idea to have the vaccines, I know this probably not-, well, it would be easy to have the vaccines whenever your at your maternity appointments-, because you get most of your appointments, and you have to get it again for your vaccine, so it would be good-, I know what though that it's just probably being a bit greedy, but that would make things a whole lot easier. "(Focus group participant 5)
Reflective Motivation	(4.1) Unnerved by the unknown (vaccine risk and safety)	- Fear of unknown impact & unknown substance	"But, as I said, damned if I did and damned if I don't because if I didn't get it and something happened and if I did get it and something happened. But I did feel sick getting it, both times, I cried whenever I got to go to them. I just thought, 'If somebody tells me so many years down the line that my child has this because of the vaccine then it's my fault.' But then, if you had got COVID or something happened during the pregnancy, I said, 'You could avoid this if you got the vaccine then. 'It's just all unknown." (Focus group participant 5).<br><br>"And I knew that taking the vaccine wasn't risk free, because we didn't have the information, but I knew that COVID from my own, not from being a nurse, just from reading myself that COVID was causing women to

			have problems with their placenta. And then the babies, some babies were dying, some babies were being born early." (Focus group participant 4).
		- Benefits vs risks uncertain or vague	"Not really. I was just asked, 'Did you have it?' But whenever you said I have it, like they never went into any information about it. I think because I'd already had my first one and just asked the doctor the second of, 'How will I get this?' And he said, 'Yes.' Like there was no information given to me, like it wasn't, 'This is what's going to happen, or it might do this.' It was just, 'Get it.'" (Focus group participant 1).<br>*[In response to the moderator follow-up prompt regarding whether risks and benefits where discussed by HCPs/midwifes, all focus group participants agreed with the above sentiment]*
	(4.2) Confidence in personal health agency	- Informed choice is important	"But I think just, in my mind, I think just opening up that dialogue with people, they don't have to get it, but they need to make an informed decision about it and be able to go and air their views without judgement. Like, say, I am worried about development, or I am worried about this, and then speak with somebody who is educated in it, who does know what they're talking about and blank out all those voices of family, friends, health professionals that think they know but don't actually know. Things like that where they can-, they might not even make the decision to take it, but they can explore the reasons why they would or would not take it."(Focus group participant 4)<br>"I think maybe-, I don't know a lot about it to be honest, but I think maybe that we should so they can make informed decisions and change things, but I know what P2's saying. Whereas you do get what you're looking for, but I would hope that the researchers would be impartial because at the end of the day, that's how we figure if something that's been effective,

		- Optimism in health choices is important**	who we’re reaching and who we’re not reaching, is through research. But I don't know a lot about it, so.” (Focus group participant 5).<br><br>“No, if you got the vaccine, you were sort of hoping you would take milder symptoms, if you did get it, then you could end up in hospital.” (Focus group participant 1).
Automatic Motivation	(5.1) Fear drives indecision<br><br>(5.2) Feeling unheard and cornered	- Worry and fear (scary stories and unknown impact)<br>- Unease and uncertainty (risks and unknown impact)<br><br>- Feeling trapped, pressurised and/or judged	“I have, whenever I had the vaccine, I had got a massive headache after my second vaccine. And I was admitted into hospital, and they treated me for a clot straight away. So, I was terrified at that stage, I thought. And of course, you do Google it, I know you shouldn't, but you do Google it. And it was like, 'Well, there's a high risk of your child coming early.' So, I was like, 'How many weeks is he?' And all of that, it was a scary thing for me, thank God, I was okay I don't know what to put it down to, maybe a massive migraine. But I would say was a result of all the stress of the unknown from the vaccines.” (Focus group participant 5).<br>“I would say so, but I am not going to tell you-, I still think I will still always be anxious, a wee bit anxious about baby's development down the line if anything comes up. The first thing that'll probably pop up into my head will be, was that a result of the COVID vaccine? But I hope that never happens, but I suppose-, don't know.” (Focus group participant 5).<br><br>“I thought that something really pregnancy specific because you feel like when you go, that you're the only pregnant person-, well I did, I was the only pregnant person in the room and the other people were looking at you and, ‘oh gosh, she's getting that and she's pregnant’. In Derry here, maybe P5 would remember as well that they had specific pregnancy days

			where they protected time for pregnant women. Unfortunately, there was a big protest outside, the anti-vaxxers gate-crashed it, but I thought that would be useful." (Focus group participant 5).
		- Lack of/need for empathy and understanding	"I think health inequalities are real and there are specific groups who are missing out on vaccines and on general health because they are in a certain group or a certain socioeconomic class or whatever. In order to help best reach people in the way that they-, we need to reach people in the way that is meaningful to them as well, that they will understand and it will be appropriate for them, that they would be interested and listen to them. Not everyone's going to be a nerd like me and go on and look at immunologists and all that there, they wouldn't find that interesting. But in a way that's meaningful to them, it will be important because they are missing out and they don't-, it is unfair. You might think that they've made that choice, but it's not necessarily true, it's just because they fit in a certain class where they maybe don't know where to look for the information or their education-, they wouldn't know what is sound research and what's not sound research and things like that, in ways that best reach people. I think they should be allowed to look at that." (Focus group participant 5).

Note. **Denotes any differences in the codes identified for focus group participants
